# Tensions experienced by student and care professionals in a learning and innovation network: a responsive evaluation using storytelling

**DOI:** 10.1016/j.ijnsa.2025.100360

**Published:** 2025-05-29

**Authors:** M.(Marjolein) Albers, R.J.J.(Robbert) Gobbens, M.(Margreet) Reitsma, H.L.G.R.(Henk) Nies, O.A.A.M.J.(Olaf) Timmermans

**Affiliations:** a Faculty of Health, Sports and Social Work, Inholland University of Apllied Sciences, De Boelelaan 1109 1081, HV, Amsterdam, the Netherlands; b Vilans, National Centre of Expertise for Long-term Care, Churchilllaan 11 3527, GV, Utrecht, the Netherlands; c Faculty of Medicine & Health Sciences, Centre for Research and Innovation in Care, University of Antwerp, Universiteitsplein 1 2610, Wilrijk, Belgium; d Department of Organization Sciences, Faculty Social Sciences, Vrije Universiteit, De Boelelaan 1105 1081, HV, Amsterdam, the Netherlands; e Research Group Healthy Region, HZ University of Applied Sciences, Edisonweg 4 4282, NW, Vlissingen, the Netherlands; f Zonnehuisgroep Amstelland, Groenelaan 7 1186, AA, Amstelveen, the Netherlands; g Faculty of Medicine and Health Sciences, Department Family Medicine and Population Health, University of Antwerp, Universiteitsplein 1 2610, Wilrijk, Belgium; h Tranzo, Tilburg University, Warandelaan 2 5037, AB, Tilburg, the Netherlands

**Keywords:** Nursing education, Healthcare innovation, Role ambiguity, Geriatric rehabilitation, Qualitative research, Quality improvement

## Abstract

**Background:**

Learning and innovation networks are collaborative initiatives between educational and healthcare institutions aimed at integrating the learning of students and care professionals and improving the quality of care through shared practices.

**Aim:**

This study aims to explore how care professionals and students experience learning and working within a learning and innovation network.

**Methods:**

Using a responsive evaluation, we collected data in four iterative phases: (1) interviews with four students and two care professionals to identify themes; (2) development of four fictional stories illustrating tensions based on interview data and practical experience; (3) focus groups with students and care professionals to validate and refine the stories; and (4) a mixed stakeholder group discussion to reflect and start to formulate improvement actions. Thematic analysis was used across all phases.

**Results:**

Participants experienced various tensions, and one of the primary challenges for both students and care professionals was to balance the time allocated between learning activities, care provision and innovation projects. Students faced role ambiguity when performing unsupervised care tasks, feeling part of the team but also vulnerable in their learner status. Care professionals struggled with time constraints and competing demands between patient care and student supervision, which sometimes led to them withdrawing from guiding students. Another tension emerged around engaging in quality improvement. While participants recognized the importance of improving care, engaging in this progress was hindered by a lack of shared understanding of optimal rehabilitation practices and unclear responsibilities regarding implementing innovation. The student-dense environment, though rich in learning potential, heightened these tensions due to differing expectations and unclear roles.

**Conclusion:**

Although learning and innovation networks offer valuable opportunities for mutual learning and improving care, they also introduce tensions that need to be acknowledged and addressed. Managing these tensions, especially around time division, role clarity and a shared vision for improving care, is essential for creating sustainable learning and innovation climates in practice.

**Contribution of the Paper**

What is already known about the topic

Students and registered nurses benefit from workplace learning.

Joint initiatives by educational and healthcare organizations have a positive effect on the competences of nurses.

What this paper adds

It suggests that student-dense wards can create rich learning environments.

It provides insight into the thoughts and feelings of students who face unclear roles - treated as both learners and full team members.

It indicates that a lack of shared vision and role clarity hinders quality improvements.

Alt-text: Unlabelled box

## 1 Introduction

Clinical practice represents the source of the knowledge and skills needed to become a competent nurse. It is through combining education and experiences that nurses develop a professional identity and grow from novices into experts (Benner, 2010). Studies showing that workplace learning makes students better prepared for their roles shed light on learning how to handle shifting situations, build relationships, act independently, and integrate theory and practice (Berndtsson et al., 2020; Hägg-Martinell et al., 2020; Tynjälä, 2008).

Both student and registered nurses may benefit from workplace learning, as learning from feedback, reflection and observing others is correlated with nurses’ perception of their levels of competence (Takase et al., 2015). Moreover, informal learning, an important element of workplace learning, can refine nursing practice through puzzling and enquiring, an iterative process of learning while nursing (Jantzen, 2019).

For years there have been joint initiatives by educational and healthcare organizations aimed at educating nurses, often driven by a desire to train more nurses in a shorter period of time or to enhance their training by reducing the theory-practice gap (Hooper et al., 2020; Peterson and Morris, 2019). Dedicated education units are examples of cooperation between universities and healthcare organizations in which a nursing ward is developed into a clinical learning environment. Faculty members and staff nurses work together to transfer classroom learning into practice and grade students on achieving learning outcomes (Glazer et al., 2011).

Studies of dedicated education units reveal positive experiences among both students and care professionals, with students being offered more learning opportunities and hands-on experiences in a positive learning environment, leading to a significant increase in specific competencies and professional attributes such as critical thinking scores (Bittner et al., 2021; Hooper et al., 2020; Rusch et al., 2018; Williams et al., 2021). Musallam et al. (2021) state that dedicated education units have a positive effect on clinical self-efficacy and confidence, teamwork and collaboration, and knowledge and competency. Moreover, these units also manage to increase the number of students in the clinical area (Hooper et al., 2020).

Dedicated education units also have a positive impact on care professionals, reporting that, thanks to the improved learning climate, they are better able to fulfil their role as preceptors and report positive learning experiences and higher satisfaction levels with students’ professional growth (Hooper et al., 2020; Williams et al., 2021). Schoening et al. (2021) state that staff nurses also benefit from staying current and experience the faculty presence as positive. Their study also points out, however, that staff regularly report having insufficient time to supervise their students.

Other collaborative initiatives between educational and healthcare organizations, originally founded in The Netherlands, include care innovation units and learning and innovation networks (Albers et al., 2021, 2024; Meijers et al., 2022; Snoeren et al., 2016). Despite their different names, the concepts of the above-mentioned units are similar and they also share characteristics with dedicated education units in terms of an effective academic-practice partnership, adaptability to diverse contexts, building nurse and faculty capacity, facilitating student learning, communicating regularly at system and unit levels, and developing nursing practice (Albers et al., 2024). A learning and innovation network comprises healthcare professionals, students and educational representatives who come together to form a learning and innovation community in nursing to integrate education, research and practice (Albers et al., 2024). The partners in the network work together on practice-oriented projects in which best practices, research evidence and client perspectives are combined to innovate and improve the quality of care (Albers et al., 2021). Such a network typically involves an educational representative in the form of a lecturer practitioner who is at the healthcare organization for at least four hours a week and engages with students and care professionals in wide-ranging activities to learn with and from each other to enhance the quality of care (Albers et al., 2024; Meijers et al., 2022). In the study by Snoeren et al. (2016), it appears that students in care innovation units are satisfied with the units' learning potential, which is formed by various interrelated and self-reinforcing affordances: co-constructive learning and working, challenging situations and activities, being given responsibility and independence, and supportive and recognizable learning structures.

An important difference between dedicated education units and care innovation units or learning and innovation networks is the emphasis on care professionals’ learning and the focus on innovation to enhance the quality of care (Albers et al., 2021, 2024). Following the definition of nursing innovation as “the encouragement of professionals to utilize their acquired knowledge and skills to creatively generate and develop new ways of working, drawing on technologies, systems, theories and associated partners/stakeholders to further enhance and evaluate (nursing) practice” (McSherry and Douglas, 2011, p. 165), it is important for nurses to innovate to keep up to date with the rapidly changing complex demands of care. This means that the educational programme for nurses should require training in innovation skills and agile competences. According to Strand and Tveit (2020), the experience of running a small-scale quality improvement project in clinical practice is well suited to equipping students with useful competences for the future. The results of their study indicate that the project made the students more confident, braver and more aware of their future role and responsibility for quality improvement in nursing practice. The authors also found that staff involvement and cooperation, when imparting new knowledge, was important to create an atmosphere of change.

However, it is rather challenging in practice for nurses to fulfil all roles equally: they must have both a production and a development orientation (Timmermans et al., 2013); they must process the information needed to execute the production process, such as on patients and planning, but also that needed to reflect on and innovate their current ways of practising to work according to the latest standards and be future proof.

In terms of learning in clinical practice, much can be gained if students, care professionals and education representatives join together in the processes of delivering and improving nursing care. There are a lot of barriers to overcome in order for this cooperation to blossom fully (Albers et al., 2024) – barriers that are mainly felt by care professionals and students, as it is challenging for them to balance continuous professional development with the demands of care delivery. The aim of this study is to explore the experiences of care staff and students in a learning and innovation network.

## 2 Method

We used a participatory action research design (Abma et al., 2019; Guba and Lincoln, 1989). The study was conducted in a learning and innovation network located in a geriatric rehabilitation ward at a nursing home in The Netherlands. The first author of this study is a lecturer practitioner affiliated with the learning and innovation network studied in this research; she teaches at the nursing school of Inholland University of Applied Sciences, conducts PhD research and dedicates half a day a week to working within the network through collaboration between educational and health organizations. By combining her roles as a researcher and a participant in the network, she is closely engaged with practice, aligning with the philosophy of participatory action research.

Box 1 **Background information on the study context**

The ward in this study has 31 places for older people following an acute or subacute decrease in function after a medical event such as a hip fracture or exacerbation of chronic obstructive pulmonary disease, and occasionally for people to regain strength after neglecting their health at home. Physical conditions are often combined with psychosocial difficulties – e.g. homeless people with a small social network of people with psychiatric disorders, but a (recent) history of alcohol or drug addiction.

In the multidisciplinary team, an elderly-care physician, three physiotherapists, an occupational therapist, nurses and nursing assistants work together. Depending on the patients’ needs, the team can be extended to include a speech therapist, a dietician, a psychologist and a social worker. As in other nursing homes, the care professionals on the ward of interest comprise some registered nurses but mostly certified nursing assistants (Vaalburg et al., 2023). Compared to other countries, the Dutch-certified nursing assistant education is rather lengthy, comprising a three-year practice-oriented course (Van Wieringen et al., 2022).

Since February 2020, eight students (four vocational, four higher education) have been assigned to the team, together with a teacher (lecturer practitioner) from Inholland University of Applied Sciences. One afternoon a week, all students, the teacher and four representatives of the nursing team (in-group members) engage in learning activities such as peer review, moral deliberation, clinical reasoning exercises, lessons from experts and short-cycle projects to improve care. Besides formal learning, many informal learning moments arise in practice when in- and out-group care professionals guide students to become nurses. Students have dedicated supervisors, but if the latter are absent, every team member is supposed to guide the students. Other important stakeholders in the learning and innovation network are the team manager and placement supervisor.

Alt-text: Unlabelled box

We used responsive evaluation, which aligns with the principles of participatory action research. The latter focuses on sequential reflection and action, carried out with and by local people (Cornwall and Jewkes, 1995), while responsive evaluation is aimed at improving interventions by aligning them more closely with practice (Van Heijster, 2022), in the knowledge that social reality is too complex to detect clear cause-effect relationships between an intervention and an outcome (Abma and Noordegraaf, 2003). Responsive evaluation is both research and an intervention, often adopting a mixed-method design that facilitates dialogue among important stakeholders, notably the voices of those less heard. It fits the ideal of participatory action research being an emancipatory process: as Guba and Lincoln (1989, p. 65) state, “one of the aims of responsive evaluation is to protect against exploitation while empowering and enfranchising [the] less powerful”. The impact of responsive evaluation is often described as a learning process, in which understanding is gained on important issues for stakeholders, leading to changes in understanding, attitude and sometimes organizational and/or individual behaviour (Van Heijster et al., 2022). Because the dialogue is not aimed at reaching consensus but rather at discovering where the differences are, it offers a better understanding of possible solutions (Niessen et al., 2009). In the context of this study (for more information about the context see Box 1), the students and care professionals are the ones who invest most time and effort into the learning and innovation network, while they are less heard in the planning and decision-making. We focus on their perspectives here.

The research was conducted in four phases that build on each other. In phase 1, we performed individual interviews with students and care professionals to retrieve important themes. In phase 2, authors MA and RG wrote four stories based on the important themes from the interviews and complemented these with their personal practical experiences. In phase 3, we exchanged and checked the stories: students and care professionals heard the stories from their own and the other group and complemented and adjusted them. Finally, in phase 4, a mixed group of involved professionals exchanged the stories and started thinking about how to resolve the issues that needed to be addressed.

### 2.1 Participants

A purposive sampling technique was used to select care professionals and students from two cohorts. They all worked or studied on the ward between September 2021 and July 2022. We excluded students and care professionals who had less than ten weeks of experience on the ward, as well as temporary staff. We spoke with new participants in each round. Participants for all rounds were invited and informed by e-mail and recruited personally by the first author.

See Appendix 1 for the demographic variables of every participant, with a note that, due to identifiability concerns, limited variables are published.

#### 2.1.1 Phase 1: interviews

We interviewed two students of vocational nursing education, two students from the Bachelor’s in nursing education and two care professionals who were in the inner circle of the network, meaning they engaged in weekly half-day gatherings. The interviews took place in a private meeting room at the workplace or online, and we randomly selected the first person to interview within each subgroup. At the end of the interview, we asked the respondent who they thought was the person with the most opposite opinion and then invited that person to do the next interview to ensure maximal variation of opinions within the snowball method (Douglas, 2022). The interview topics were as follows.

a) The attractiveness of the profession generally and of geriatric rehabilitation specifically.
b) Experiences with the LIN: influence on the respondent, team and patients.
c) Experiences with learning: what did people learn, how and from whom? How could learning be improved?
d) Experiences with innovation and the process of improving quality of care.
e) Major ingredients if they were to start their own LIN.

#### 2.1.2 Phase 2: writing stories

A narrative or story (the terms are usually used interchangeably) is a unique type of discourse characterized primarily by a teller (perspective), characters, plot, endpoint and sequence (Riessman, 2008). We used a tensional approach (Hong et al., 2017) in which we explored key moments of tension characterized by unease and discomfort. According to Senge (2006), tension is an essential part of the learning process; it can motivate individuals and teams to take action to close the gap between the current reality and where the person or organization aspires to be. For the focus groups, the first author (MA) wrote four stories based on recurrent difficulties and major themes mentioned in the interviews, complemented by experience in practice to make the stories feel even more real, but also more anonymous. RG checked the stories for major themes and comprehensiveness. All stories were written in the first person and pictured a situation, a perceived tension that had to be resolved and a solution. We wrote one story for the theme of learning from the student perspective and one from the care professional's perspective, along with one for the theme of innovation from the student perspective and one from the care professional's perspective.

#### 2.1.3 Phase 3: homogeneous focus groups

We organized two focus groups: one for students and one for care professionals, using convenience sampling. Individuals were working on the ward at that time but could leave it for 90 min. None of the participants were from the interviews. The focus group with the students was performed on a day that all students were on the ward. In the focus group with the care professionals, three team members participated: two from the inner and one from the outer circle. The aim was to learn others’ perspectives, reflect on their own, and sharpen the stories to be used in the combined focus group in phase four. The participants read each story and were asked whether they could relate to it. They were encouraged to tell their own stories and asked about their feelings; they were also asked how they felt the tension from the stories could be resolved. At the end, the group decided whether something had to be added or removed from the original story, leading to slight changes.

#### 2.1.4 Phase 4: mixed focus group

In this focus group, we purposively invited a mix of stakeholders (see Table 1) because we wanted all to hear the stories of the students and care staff. We chose first to read the stories on learning from both students’ and care professionals' perspectives, then asked for responses. Could they relate to the story? What was their perspective on the situation? After that, the innovation stories were read, and the same questions were asked. At the end of the focus group, we asked what had to be done to improve learning and innovation on the ward.

**Table 1:** Participants.

Phase	Interviews	Homogeneous focus group	Mixed focus group
**Participants**	Two students of vocational nursing education Two students of higher nursing education Two care professionals from the inner circle	**Focus group students** Three students of vocational nursing education Three students of higher nursing education **Focus group care professionals** Two care professionals from the inner circle (involved in weekly gatherings) One care professional from the outer circle (not involved in weekly gatherings)	One student One care professional One quality improvement nurse One team manager One placement supervisor One lecturer practitioner

To detect representative citations on the major tensions and their stories, we performed a thematic analysis (Clarke and Braun, 2013). Author MA searched for tensions in the transcripts of the interviews, stories and focus groups, created a code list and coded all outcomes. RG coded all texts with the code list from MA and checked whether more tensions could be found. OT coded the outcome of the final focus group and stories using the code list from MA and checked whether more tensions could be found. After the three researchers had coded the outcomes separately, differences and similarities were discussed.

### 2.2 Rigour

To demonstrate the rigour of the qualitative methodology, we chose to use the four dimensions described by Forero et al. (2018) based on Lincoln and Guba’s (1985) criteria for qualitative research (see Table 2).

**Table 2:** Four-dimension criteria for qualitative research.

Rigour criteria	Purpose	Strategies applied in our study to achieve rigour
Credibility	To establish that the results are true, credible and believable	The different backgrounds of the researchers (MA works in a learning and innovation network in practice, RG and OT are researchers in another organization) provided a mix of high engagement and expertise within the setting and an outside view. The first author has prolonged engagement with the setting, having been a lecturer practitioner for two years. The third focus group session was moderated by an outside experienced female interviewer who was familiar with the concept of the network and geriatric rehabilitation in another organization. The three phases of the study that build on one another made it possible to check the stories and researchers’ interpretations. We did not return the transcripts to participants for comment. The semi-structured interview procedures allowed focus and flexibility during the interviews. We asked the respondents from the interviews to name the colleague with the most opposite opinion to invite them to attend the next interview to make sure we got the widest range of views. The first author kept a log of personal impressions during the recruitment of respondents and her thoughts and feelings during the interviews and focus groups. We arranged a special meeting room outside the ward for the focus groups and a meeting room or an online meeting for the interviews. During the interviews and focus groups there were no others in the room besides the participants and the first researcher or moderator. Two interviews were audio-recorded. The online interviews were visually recorded, as were all focus groups.
Dependability	To ensure the findings of this qualitative inquiry were repeatable if the inquiry occurred within a similar cohort of participants, codes and context.	We produced a detailed record of the data collection process. We documented changes to the stories based on the focus groups. All the steps in coding the data and identification of key concepts were agreed upon by members, MA, RG and OT. The coding material was often put away and retrieved after considerable time to see whether the codes were still sufficient.
Confirmability	To extend confidence that the results could be confirmed or corroborated by other researchers	We used a combination of interviews and focus groups, and the experiences of the researcher to complement the story, enabling methodological triangulation. Reflexivity: we organized two meetings with all authors to discuss interpretations of the tensions found and checked whether the descriptions were in line with the outcomes. We looked for evidence of tensions by comparing them with previous studies.
Transferability	To extend the degree to which the results can be generalized or transferred to other contexts or settings	Data saturation: we interviewed almost all students who were part of the network at that time. The same applied to the quality improvement nurse, team manager, placement supervisor and lecturer practitioner.

### 2.3 Ethical considerations

This study has been reviewed by the Research Ethic Review Committee of the Faculty of Social Sciences of the Vrije Universiteit, reference number ERB/20–02–01. The committee declared that the study complied with the faculty guidelines. All respondents were invited via email and/or personally and informed about the study, with all participating voluntarily and declaring informed consent.

## 3 Results

In the phase 1 interviews with students and care professionals, several main themes on learning and working in a learning and innovation network emerged. The themes were similar for both groups, albeit seen from different perspectives – in particular, the experiences around innovations and the participation of the rest of the team. The main advantages and/or tensions from the students’ and caregivers’ perspectives are described in Table 3.

**Table 3:** Results of the interviews.

	Advantages and barriers	Tensions
Student perspective	• The advantage of being with a group of peers: students can relate to and learn from each other. • Didactical work forms enabling learning processes: Due to the learning and innovation network on this ward, care professionals are open to guiding students. • Shortage of staff as a barrier to learning.	• The tension of autonomy: the opportunity and trust from the team to work autonomously versus the need to learn under supervision. • The tension of the position in the team: when do I choose my own learning process versus when do I help the team with care delivery in times of staff shortages or in improving care? • The tension between investing time in formal assignments from educational organizations or the quality improvement project and informal learning.
Care professional perspective	• The advantage of learning with and from students. • The advantage for patients because they receive more attention now the students are here. • Shortage of staff as a barrier to enjoying the process.	• The difficulty of guiding so many students in limited time, which raises the question of taking a role as a guide for students or as a nursing professional. • The tension in guiding: who takes the lead in the student’s learning process? Is that the responsibility of the student or the supervisor? • The tension between investing limited time in the direct delivery of care or improving quality of care on a *meso* level. • Tensions in team members’ openness to change.

Based on the themes and anecdotes from the interviews or experiences in practice, fictional reality-based stories were written in phase 2. The group of students could relate closely to the story of the fictional first person. They recognized her story and shared their own similar anecdotes. As a result of the phase 3 focus group with in-group students, two sentences were inserted to sharpen the story: we added the student’s focus on formal assessment and elaborated on challenging oneself. In the phase 3 focus group with care professionals, the staff also related to the fictional nurse’s story; however, they also came up with some new considerations. We changed the text of the care professional’s perspective on learning and added the following elements: students seem very busy with various formal assessments, so there is less time to learn in practice; care professionals sometimes feel they become obsolete when there are so many students; and some colleagues lack the competence to shape the guidance of students (see appendix 2 for the four stories).

**Fig. 1:**
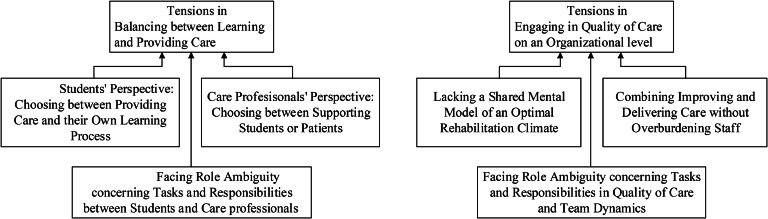
Tensions in Learning and Innovation Networks.

After the phase 4 mixed focus groups had been conducted and all data analysed by the three researchers (i.e. interview and focus group transcriptions as well as the stories), we detected two major themes, each containing three subthemes (see Fig. 1).

### 3.1 Tensions in balancing between learning and providing care

Both students and care professionals have difficulties balancing between engaging in and guiding learning combined with providing care. On a daily basis they have to choose where to focus their effort and reflect on their role.

#### 3.1.1 From the students’ perspective: the continual choosing between providing care and their own learning process

After the induction period when students are familiar with the patients, colleagues and processes on the ward, the workload is distributed between care professionals and students.

Because you can do it yourself, you will do it. (Student 11)

On the one hand, working autonomously encourages students to have their own caseload and prepares them for their future work; on the other hand, the independent provision of care on their own may prevent students from growing while learning from feedback from others and from new situations.

I'm proud that I took on the challenge, that I did the intake of the new patient myself, because a situation like that gives you a push. (Student 3)

It was difficult to choose, because I actually wanted to watch the wound treatment, that was also my learning goal, but I didn't want to leave that lady and the team behind either. (Student 1)

Asking for attention and time from care professionals is often difficult.

In the morning I explained my learning goals, but then during the day my supervisor says that she is too busy to give feedback. (Student 4)

#### 3.1.2 From the care professionals’ perspective: the continual choosing between supporting students or patients

Care professionals mostly enjoy guiding students on the ward: they feel the responsibility of educating and inspiring young people to join the nursing profession and enjoy demonstrating their expertise to the juniors.

Yes, I really thought it was one of the best things there was at work. Supervising students and sitting in class and just looking at the department a little more critically together with young people. (Care professional 2)

Supervising students, however, can be rather demanding in terms of time as well as planning, organization and guiding skills, especially in a learning and innovation network where there is a large number of students.

To work on a ward like this requires even more from people … I have the idea that is not always feasible for everyone to keep that overview completely. (Care professional 3)

Do I have time to watch anyone? And where do I find the time to sign all kinds of forms and read assignments? (Care professional 4)

When circumstances are far from ideal because of a shortage of staff or extra work, like there was during the Covid pandemic, guiding students is one of the first activities to be eliminated: the provision and coordination of care always comes first.

And there are so many things that need to be done in the morning – the care, the doctor's visits, the multidisciplinary meetings, all that kind of stuff […] The students can go their own way. (Care professional 1)

One of the care professionals in the focus group concluded:

My priority is the ward: the patients you want to help. I understand that there are students and that you have to invest in them. But you can't leave people to their fate. Yes, it gives mixed feelings. (Care professional 3)

### 3.2 Facing role ambiguity concerning tasks and responsibilities between students and care professionals

The tensions mentioned above, combined with a lack of time, structure and skills, often lead to tension in the student’s position in a team: can one consider a student a colleague and expect them to have a considerable caseload and give them a voice once properly inducted, or, because of the nature of the coming and going of the students and the fact that it is the group of students that are assessed, should they be seen as part of the team?

On the one hand, students often feel part of the workforce:

That was about my third week and there was really no one else who could make time to do the intake of the new patient. So, when I was asked “do you want to do it?”, I felt like I couldn't say no. (Student 5)

On the other hand, they do not always feel like they are up for it:

The problem is not that you have to work harder, it is the responsibility that makes it stressful. (Student 8)

Care professionals also see students as part of the team – sometimes to bridge gaps in staffing, sometimes to give some extra attention to patients:

Sometimes I hear from others that students are considered as workforce. (Team manager)

I think the students did a lot of good for the patients, they really had time for them, and they really took the time for the rehabilitation process. (Care professional 2)

In interchangeable learning situations, students can be seen by team members as equals:

The peer review sessions were interesting to be part of, you just come to a conclusion together: gosh, how could we better guide a patient in rehabilitation in the department? (Care professional 1)

On the other hand, students sometimes feel they are seen as less than colleagues:

As a student, you are quickly seen as a bit, I wouldn't say less, but more like: “I have had my experience and my diploma for so long, You're still learning. Who are you to tell me something?” While you can learn from each other quite well. (Student 11)

Because care professionals have a say in assessing whether a student does or does not pass their exam, it can be considered inevitable that students are unequal:

As a student you also have a bit of a vulnerable position in a team. (Student 11)

Nevertheless, there are also care professionals who view students as equally competent and sometimes having an even bigger voice than they do themselves, possibly because of their large number:

Well, we were once in a team meeting and the students had more input than we did, we were such a small group. (Care professional 1)

Overall, care professionals don’t always know what to expect from students regarding their competences, their possible workload and ownership of their learning process:

I just think we need more clarity about what is the role of the student and what is the role of us: what they can do for us and what we can do for them. (Care professional 3)

### 3.3 Tensions in engaging in quality of care on an organizational level

Besides the tensions in the learning process and in providing care, there are also tensions related to engaging in improving quality of care.

#### 3.3.1 Lacking a shared mental model of an optimal rehabilitation climate

In the context of geriatric rehabilitation care, team members are not aligned in offering the best possible rehabilitation climate.

One colleague cares with her hands “behind her back”; she stimulates the patient to be independent and self-reliant. The other colleague takes over all the actions the patients can undertake themselves. (Team manager)

It's good that you start thinking together again: what do we stand for? What are we good at together? (Quality improvement nurse)

#### 3.3.2 Combining improving and delivering care without overburdening staff

In general, the team know there is room for enhancing the quality of care they deliver, and the limited actions taken seem to be partly due to the high workload.

There are conversations about so many subjects, so: how are you going to do that in a way that you don't burden people with all kinds of new ideas? (Team manager)

I also think that the Covid pandemic didn't help because everyone's focus was really elsewhere. (Care professional 3)

But respondents also sense a lack of openness to change among their colleagues:

I also don't really feel that people are open to change. When they hear it, they all say they know it. But no one does anything with it, so we keep a status quo. (Student 5)

If you work somewhere for a long time, at some point you get institutionalized: this is how it works, this is how we do it and nothing else. (Care professional 3)

#### 3.3.3 Facing role ambiguity concerning tasks and responsibilities in quality of care and team dynamics

Both care professionals and nursing students view delivering and coordinating care as their main job.

Those quality improvement projects did not appeal to me, I just want to work with patients; it is not your job to improve care. (Student 4)

So the students also think it is not interesting to work on quality of care? (Care professional 4)

Working on and implementing improvements in care through short-cycle projects seems to be the territory of the students and lecturer practitioner, and sometimes the manager or nurse leader, and not so much a responsibility of the team itself.

The owners were actually the students, me and the teachers. I think the ward itself should have been the owner of it. The employees should be involved. (Care professional 2)

The manager can indicate that it is important that my colleagues comply with this new procedure. Look, if I say that, they're not going to do that. (Care professional 1)

But not all these participants feel up to involving team members to induce a positive change:

You feel bad that you have to point out to them how to do it differently after so many years, and I'm here just for so many months. (Student 7)

But that's frustrating […] if you want to improve things that could give relief, for example, and you can't get that off the ground, it can give a lot of frustration. (Quality improvement nurse)

I don't want to be the bogeyman all the time. (Team manager)

If role definitions and shared goals are not clear, it is challenging to find a productive base in day-to-day practice.

## 4 Discussion

This study sought to explore how care staff and students experience learning and working in a learning and innovation network. Through interviews and focus groups with stakeholders with usually the quietest voice (i.e. student nurses and care professionals), we found several tensions they have to deal with daily while working and learning in a learning and innovation network. Most of the tensions we found are also supported by other studies.

Both students and care professionals are involved daily in making choices about how to divide their time between providing care to patients and slowing down care delivery in order to come to deeper or wider learning. Both groups have their own issues regarding this tension: students have to choose between their own learning process and helping in daily care delivery, while professionals have to divide their time between the large number of students and patients. When, due to circumstances or skills, supervising students is hard to combine with regular work, guiding them is the first thing to be eliminated by care professionals.

The tensions students experience in placements are also supported by other studies, which describe the stress nursing students experience due to a combination of academic pressures and clinical practice (Admi et al., 2018; Olvera Alvarez et al., 2019). Students experience stress when they feel insufficiently prepared to cope with knowledge and skill demands, experience conflicts between professional beliefs and the reality in practice (Admi et al., 2018), and feel they are constantly being observed and evaluated (Bhurtun et al., 2019).

With regard to the tension found in our study related to the continual choosing between supporting students or patients, Browning and Pront (2015) and Harrison-White and Owens (2018) describe the demanding expectations of nurses in their dual roles of supervising students and providing care, which are even more evident on student-dense wards (Bøe and Debesay, 2021). Care professionals in learning and innovation networks are expected to experience a higher workload related to supervising students on a student-dense ward but a lower patient workload once the students have been properly induced. Whether that appeals to them and all involved care professionals are well suited is, however, questionable. This is important because nurses’ restricted time or competences affect the learning possibilities and safety of nursing students (Motsaanaka et al., 2020), as they are the “gatekeepers to learning possibilities” (Harrison-White and Owens, 2018, p. 80).

As regards the role ambiguity or role uncertainty found in our study, several studies describe similar tensions. The perception of students as future colleagues positively influences satisfaction with clinical practice (Atack et al., 2000; Papastavrou et al., 2016); students experience mutual courtesy and respect, open communication, and shared knowledge and decision-making. Nursing staff’s perception of student nurses as being sometimes workers and sometimes learners (Galletta et al., 2017) can also lead to stress due to role uncertainty and role conflict and the challenge in bridging the gap between undergraduate educational curricula and workplace expectations (Rudman and Gustavsson, 2012; Wu and Norman, 2006). This stress is expected, on the one hand, to be higher for students in learning and innovation networks, because they are given more responsibility on these student-dense wards, but on the other hand, the stress experienced may be lower because the lecturer practitioner can, through wide-ranging work forms, help to integrate formal and informal learning (Albers et al., 2024). Furthermore, in learning and innovation networks, this ambiguity applies not only to students but to care professionals as well. One moment you are a student’s supervisor and the next moment, due to the emphasis on reciprocal learning in the network, you are a fellow learner.

When it comes to improving the quality of care, students and care professionals know they have to deliver qualitative person-centred care on a personal level, but they do not have a shared mental model of the best rehabilitation climate or their role in improving care on an organizational level.

Senge (2006) describes the importance of a shared mental model: they help teams align their perspectives, foster collective understanding and coordinate actions. The tension caused by the lack of this shared mental model of an optimal rehabilitation climate might not only be present in this ward but also prevalent in other wards in the relatively new line of work in geriatric rehabilitation. Tijsen et al. (2023) describe the considerable differences in the interpretation of rehabilitation generally and in the execution of a challenging rehabilitation environment specifically. With the different backgrounds of the team members ranging from working for years in residential care to those with, for example, experience on an intensive care unit, and neither being specialized in geriatric rehabilitation, it is challenging to develop a shared mental model of an optimal rehabilitation climate. If such models are outdated or unexamined, however, they can become barriers to innovation and learning (Senge, 2006). Investing time and effort would therefore be advantageous because this can enhance team performance (Mathieu et al., 2000). It is, however, quite challenging in practice to combine improving and delivering care without overburdening staff (Timmermans et al., 2013), and it requires a lot from the staff to include eight new student team members, with their own experiences, every semester in these mental models.

Another tension that appeared in this study was the role ambiguity of both students and care professionals concerning tasks and responsibilities in quality of care, as well as the responsibilities in team dynamics. In a systematic review on innovation in healthcare, Haring et al. (2022) identified a total of 42 tensions across nine categories, organizing tensions being the most prevalent. The review indicated that innovations in healthcare are impaired by conflicts among contradictory elements such as working cultures and convictions within the organizational and regulatory context. The authors found that the majority of tensions occurred during the implementation phase of innovations, when a multitude of parallel activities and tasks demand attention and resources. This is comparable to the tension students and care professionals experience in this study when they feel they have to engage in care delivery, learning and improving all at the same time. The study by Renju et al. (2010) suggests that if the new responsibilities of workers conflict with their qualifications and (perceived) experience, role ambiguity might occur. This supports our finding that when job expectations, responsibilities and goals lack clarity or do not fit one’s competences, tension occurs, which makes it hard to find a productive base for improvement.

### 4.1 Recommendations

Although often negatively connoted, tension can be seen as key to change (Senge, 2006). This creative tension, if managed well, can be a positive force because it motivates individuals and teams to take action to close the gap between the desirable and the current situation. To harness creative tension effectively, organizations need to be clear about their vision while being honest about their current reality. By embracing and managing tensions, organizations can sustain their momentum towards continuous learning and improvement.

Pryse et al. (2020) identified important parameters for implementing a dedicated education unit successfully, among which a clearly defined vision and goals need to be explained to all, and designated resources are needed for attending to staff and student scheduling requirements. Albers et al. (2024) show that support among all stakeholders, a shared vision and goals, and a facilitating support system are important, as are the characteristics of the participants, in terms of both quantity and competences, and their openness to learning.

Based on the results and aforementioned literature, we recommend investing in team meetings to define the vision and goals in three areas: what we consider an optimal rehabilitation climate; what we see as an optimal learning climate; and what we view as an optimal innovation climate, possibly preceded by the question of whether the team wants to continue being part of a learning and innovation network, as their support is critical. It would be interesting to consider doing this through participatory action research and also include students and the lecturer practitioner in it. It is, however, important that a learning and innovating climate is seen in the light of the entire team and not limited to students. Theories on team learning (Decuyper et al., 2010; Timmermans et al., 2013) might provide the inspiration to define the vision and, later in the process, the desirable situation. We are aware that this may take a considerable amount of time, which can be ill afforded. We believe, however, that this investment pays. If it is the desire of the organization to continue as a learning and innovation network, it is required to provide team members with the time to meet, and offer a structure to enhance clarity. After setting the vision and goals, we would highly recommend clarifying roles in the process.

### 4.2 Limitations

An important contextual factor in both the implementation of the network and the responsive evaluation is that it all took place during the Covid-19 pandemic. This made it more difficult to meet because in-person gatherings were largely forbidden and increased the workload. Many patients deteriorated rapidly, isolation and hygienic measures took a lot of time, and there was a high sickness rate and high turnover among personnel. At the time of the study, it was possible to organize meetings again; however, the turnover among personnel was still high.

Furthermore, the research positionality of the first author needs to be mentioned. The advantage of participatory action research and combining research and working in practice is that a researcher is closely related to the subject and the participants (Kitagawa, 2023). The downside can be that, because of the researching, coaching and assessing role in the learning activities of (some of the) students, which might create the issue of power imbalance (MacDonald, 2012), the retrieved data can be influenced. In designing this study, we made some choices aimed at reducing this factor as much as possible – for example, by inviting students who had already finished their placement or who were not dependent on the author for their grades.

It could also have been the case that care staff were not completely frank because their colleagues and the first author were with them. Using a mixture of individual interviews and stories of a fictional person one could relate to, we tried to reduce that limitation as much as possible. The recruitment of out-group care professionals (team members who do not engage in weekly gatherings) had its difficulties: emails were not answered by some of the care professionals, and one planned focus group could not take place because the invited individuals were not there.

The positive side of a narrative and group method was that hearing each other’s stories made behaviour, thoughts and feelings more insightful. The respondents experienced the stories as real; indeed, we had to remind them several times that they were fictional. The care professionals, for example, were very interested in the students’ perspectives and immediately wanted to change the way they worked after hearing their side. This aligns with the principles of participatory action research: emancipating, contextual and seeing research as an intervention (Guba and Lincoln, 1989). However, we did not invite students and care professionals to adopt a role as co-researchers (Abma et al., 2019). It would be interesting to convince them to become co-researchers to improve the learning and innovation network.

Future research could focus on other professionals in the network, such as the lecturer practitioner. We also recommend that future research highlights the perspectives of the team members who are not in the inner group of the network, those who do not attend weekly meetings or engage in learning activities, but who are important in implementing new findings.

## 5 Conclusion

Nursing students and staff and their support system have positive experiences with working and learning in a learning and innovation network: students support each other and are supported in their learning by care professionals, and the latter can learn with and from students enabled by wide-ranging didactical work forms, and because of the large number of students, the patients can receive more attention. Notwithstanding, there are also tensions inflicted by the student-dense learning and innovation network.

Students and care professionals are involved daily in making choices about how to divide time: students have to choose between their own learning process and helping out with daily care, while professionals have to divide their time between their students and patients. When, due to circumstances or a lack of skills, guiding students is hard to combine with regular work, the guiding of students is the first thing that is eliminated by care professionals, leading to situations whereby students care for patients without supervision and can feel like part of the workforce, giving rise to role ambiguity.

There are also tensions related to engaging in the improvement of quality of care. Students and care professionals know they have to deliver qualitative person-centred care on a personal level but they lack a shared mental model of the best rehabilitation climate. Furthermore, the responsibilities of students, care staff and other professionals regarding the implementation of innovations on an organizational level are unclear. Combined with time limitations, suggestions for improvement do not find a productive base.

## Ethical statement

This study has been reviewed by the Research Ethic Review Committee of the Faculty of Social Sciences of the Vrije Universiteit, reference number ERB/20-02-01. The committee declared that the study complies with the guidelines of the faculty. All respondents were invited through email and personally and informed about the study. They all participated voluntarily and provided informed consent.

## Funding statement

This research is funded by ZonMw within the programme ‘Kwaliteit van Zorg: Versnellen, verbreden, vernieuwen’ nr 516022517.

**Data Statement:** The data that support the findings of this study are available from the corresponding author, [MA], upon reasonable request.

## CRediT authorship contribution statement

**M.(Marjolein) Albers:** Writing – review & editing, Writing – original draft, Methodology, Formal analysis, Data curation. **R.J.J.(Robbert) Gobbens:** Writing – review & editing, Methodology, Funding acquisition, Formal analysis. **M.(Margreet) Reitsma:** Writing – review & editing, Methodology. **H.L.G.R.(Henk) Nies:** Writing – review & editing, Supervision, Methodology. **O.A.A.M.J.(Olaf) Timmermans:** Writing – review & editing, Validation, Methodology, Formal analysis.

## Declaration of competing interest

The authors declare that they have no known competing financial interests or personal relationships that could have appeared to influence the work reported in this paper.

## Appendix Supplementary materials

Image, application 1

Image, application 2
