# Supplementary material for: Tensions experienced by student and care professionals in a learning and innovation network: a responsive evaluation using storytelling
Source: Int J Nurs Stud Adv. 2025 May 29;9:100360. doi: 10.1016/j.ijnsa.2025.100360 (PMC12173126; doi:10.1016/j.ijnsa.2025.100360)
Supplement: Supplementary file 1 [file mmc1.docx]

Appendix 1:

Demographic data of participants:

| Referral | Participated in | Position within the unit | Age range |
| --- | --- | --- | --- |
| Student 1 | Individual interview | Bachelor student | 15-25 y |
| Student 2 | Individual interview | Bachelor student | 15-25 y |
| Student 3 | Individual interview | Vocational student | 15-25 y |
| Student 4 | Individual interview | Vocational student | 15-25 y |
| Student 7 | Student focus group | Vocational student | 15-25 y |
| Student 6 | Student focus group | Vocational student | 15-25 y |
| Student 5 | Student focus group | Bachelor student | 15-25 y |
| Student 8 | Student focus group | Bachelor student | 15-25 y |
| Student 9 | Student focus group | Bachelor student | 15-25 y |
| Student 10 | Student focus group | Vocational student | 15-25 y |
| Student 11 | Mixed focus group | Bachelor student | 15-25 y |
| Care professional 1 | Individual interview | RN | 55-65 y |
| Care professional 2 | Individual interview | RN | 25-35 y |
| Care professional 3 | Care professional focus group | Nursing assistant | 55-65 y |
| Care professional 4 | Care professional focus group | Nursing assistant | 45-55 y |
| Care professional 5 | Care professional focus group | RN | 45-55 y |
| Care professional 6 | Mixed focus group | Nursing assistant | 25-35 y |
| Team Manager | Mixed focus group | Team Manager | 45-55 y |
| Quality improvement nurse | Mixed focus group | Quality improvement nurse | 35-45 y |
| Lecturer Practitioner | Mixed focus group | Lecturer Practitioner | 35-45 y |
