# Supplementary material for: Tensions experienced by student and care professionals in a learning and innovation network: a responsive evaluation using storytelling
Source: Int J Nurs Stud Adv. 2025 May 29;9:100360. doi: 10.1016/j.ijnsa.2025.100360 (PMC12173126; doi:10.1016/j.ijnsa.2025.100360)
Supplement: Supplementary file 2 [file mmc2.docx]

**Appendix 2 : Four stories on learning and innovating from student and care professional perspective**

**Story 1: Student Perspective on the Theme of Learning**

Learning at this internship usually goes well. I learn a lot from others, including a lot from fellow students. I have a lot of freedom to provide care independently. Yet I am also regularly not happy. For example, someone had to be admitted to the ward once. That lady had been waiting for three hours for the intake interview, but no one got around to it, so they asked me. I had already watched an intake interview three times but had never done it myself, so that was quite exciting. But the main reason I didn't want to do it was because the wound nurse came by for the weekly round. She was going to visit the patient I often nurse and so I had a number of questions for her. It was difficult to choose, because I actually wanted to watch the wound treatment—that was also my learning goal, but yes, I didn't want to leave that lady and the team behind.

In the end, I asked a fellow student if she wanted to ask my questions to the wound nurse and that we would discuss it at a later time. That went fine. It is very nice to work with so many students in one department. We can also take the time to explain things to each other.

Unfortunately, the intake interview went pretty badly. The lady and her partner had been waiting for so long that they were already a bit grumpy and they also started asking me all kinds of complicated questions about the forms. I knew little: no one had ever explained that to me before. I persevered as best I could and called in someone from the department for the last questions. But it didn't feel good. Moreover, it did not provide me with any feedback or sign-off moment.

I eventually brought up this incident at a peer review meeting. On the one hand, I'm proud that I took on the challenge, that I did the recording myself, because a situation like that gives you a push. On the other hand, I don't think I should have done it, because no one was watching. It was not only complicated for me, but it also caused dissatisfaction for the patient. But yes ..... Maybe she'd still be there until the evening staff came.

**Story 2: Care Professional Perspective on the Theme of Learning**

It was seven o'clock on Tuesday morning, I was the coordinator (*dagoudste*) that day. When I walked into the ward, I heard it: what a noise! The entire nursing station was full, ready for the patient handover. On Tuesdays, almost all students are always there and we have our regular multidisciplinary patient discussion. I am also supposed to supervise students. I really enjoy that one-on-one, but on a day like this I don't get an overview: who wants to learn what? Who can do what independently? Do I even have time to watch anyone? And where do I find the time to sign all kinds of forms and read assignments? Today we had only three permanent employees to guide six students. Because I know from other colleagues that they prefer not to come to work on a day when the students are there—it is far too busy and they also wonder what the point of their presence is when there are already so many hands for care. Then it becomes chaotic. We don't manage to bring structure to it, even if we have already made agreements about it.

Don't get me wrong: I like that the students are there, because the patients get more attention and get out of bed faster—at least, if they are not mainly busy with their assignments. But at such a moment, I prefer to retreat and read reports before the patient handover. After all, I'm just there for the patients. That's my job as a nurse.

In the end, I took a student under my wing, a beginner to whom I could still teach a lot, and together we looked at wound care. She taught me to be a bit more handy with the reporting system: I'm just not very good at that. The other five students picked up their own work. I can only hope that went well.

**Story 3: Student Perspective on the Theme of Innovation**

We sat together again on a Wednesday afternoon—all the students, the teacher and this time one employee. On the agenda was ‘working on the quality project’ and I really didn't have the energy for it. I had two other assignments for school that had to be handed in the following week and that I still had to work on. And the project is so slow: we're not taking action.

On the one hand, I understand that dealing with overarching tasks such as the quality project is part of it...; but for me, the profession of nurse is mainly about direct care to the patient. After all, 90 per cent of your work is aimed at supporting patients on a daily basis.

Good... I got over it. I went back to looking for alternative reporting methods to the ones that are currently being used. When we presented our ideas to the other team members as a project group in the team room, I noticed that the team was not really open to it. On the one hand, they seemed to be under a high workload, and then we came to tell them what was not going well. On the other hand, I also had the feeling that they didn't want to make an effort to change the way they worked. Well, that's where it stops for me: what would I do this for? Then I focused on my own assignment again, and I thought: I'll be gone soon anyway. Figure it out for myself.

**Story 4: Care Professional Perspective on the Theme of Innovation**

It was Wednesday afternoon. I had been sitting in the LIN room on the ground floor for an hour in the weekly meeting with students and a teacher, but in my head I was actually in the department upstairs. The case discussion about Mrs. O that we just did was interesting. I did get some ideas there about how we could approach her differently. I would actually have liked to try it out right away. Moreover, they were understaffed in the ward and Mr. P would soon be picked up by the ambulance. I hoped they gave the correct medication printout. I also thought that others thought of me, she's sitting there on her ass for a while. They have to work twice as hard when we go down, with two employees and the students. There is no substitute for us when we leave the department—the staffing is not in order.

But I was sitting here.... And we talked about the improvement project that is about 'better matching the patient's rehabilitation goals'. It was going so slowly. However, I could see that we can improve a lot, but it was so difficult to get it off the ground. One week we make agreements that more than half of them don't keep, so we start all over again the next week. And if we do come up with something concrete and then share it with the rest of the team, the response is not so enthusiastic, to say the least. It seems like they don't want anything to change. Pretty frustrating. What are you doing it for?

I just went upstairs during the next break and hoped that the students would continue with it: it’s good for their learning process. And I went to Mrs. O, and that approach advice worked. Still a good day!
